# Hepatitis E Epidemic, Biratnagar, Nepal, 2014

**DOI:** 10.3201/eid2104.141512

**Published:** 2015-04

**Authors:** Ananta Shrestha, Thupten K. Lama, Sneha Karki, Deepak R. Sigdel, Utsav Rai, Shyam K. Rauniyar, Mamun Al-Mahtab, Kazuaki Takahashi, Masahiro Arai, Sheikh M.F. Akbar, Shunji Mishiro

**Affiliations:** Liver Foundation Nepal, Kathmandu, Nepal (A. Shrestha);; Civil Service Hospital, Kathmandu (T.K. Lama, S. Karki);; Koshi Zonal Hospital, Biratnagar, Nepal (D.R. Sigdel, U. Rai, S.K. Rauniyar);; Bangbandhu Sheikh Mujib Medical University, Dhaka, Bangladesh (M. Al-Mahtab);; Toshiba General Hospital, Tokyo, Japan (K. Takahashi, M. Arai, S.M.F. Akbar, S. Mishiro)

**Keywords:** hepatitis E virus, HEV, viruses, epidemic, acute icteric hepatitis E, open reading frame 2, phylogenetic analysis, water, Biratnagar, Nepal

**To the Editor:** We report a recent epidemic of hepatitis E in Biratnagar, Nepal. During the third week of April 2014, a total of 11 patients with acute jaundice came to hospitals in Biratnagar. IgM against hepatitis E virus (HEV) was detected in serum samples from all 11 patients. During the next 7 weeks, 1,861 patients with acute jaundice came to the outpatient departments of 2 of 5 large hospitals in Biratnagar; 123 patients were admitted to these 2 hospitals.

Registries at these 2 hospitals indicated that 2 patients with acute jaundice came to these hospitals on April 14. On April 28; May 5, 12, 19, and 26; and June 2, 9, 16, 23, and 30, the number of patients with acute jaundice who came to these 2 hospitals were 42, 67, 58, 69, 48, 21, 5, 3, 1, and 0, respectively. Registries showed that this increased frequency of acute jaundice lasted until the end of May 2014, when it began to decrease and reached near zero by the first week of July. In addition, unusually large numbers of patients with acute jaundice came to 25 smaller private health care facilities in Biratnagar during April–May 2014.

The Private and Boarding Schools’ Organization of Nepal closed 80 schools in Biratnagar and surrounding areas during the second week of May 2014 because of risk for disease transmission (*1*). The Biratnagar Zonal Health Authority and National Health Authority of Nepal issued special alerts by mass media regarding jaundice after the third week of April and advised using boiled water for consumption (*2*).

Registries of major hospitals, smaller health clinics, and private physicians indicated that ≈7,000 patients were affected during this epidemic. Fourteen patients died; these deaths occurred in Kathmandu, the capital of Nepal, or in different cities in India after these patients were transferred there for better treatment. Fifty pregnant women had acute jaundice, but none of these women died.

The epidemic was presumed to be caused by consumption of contaminated water (*3*). In February and March 2014, water and sewerage pipelines were damaged in different areas of Biratnagar during construction and repair of roads. A survey conducted by the Department of Community Medicine, B.P. Koirala Institute of Health (Dharan, Nepal), found high levels of coliform bacteria in water supplies from different areas in Biratnagar during the epidemic. Tap water also looked cloudy and visibly contaminated (*3*).

To obtain more information about the epidemic, the incidence of acute jaundice was determined for 656 prisoners and 75 security personnel at the Biratnagar Jail. The study protocol was approved by the Liver Foundation Nepal. Informed consent was not obtained because identity of patient samples remained anonymous. Acute jaundice was detected among 30 (4.6%) prisoners and 4 (5.3%) security personnel. The same source of consumable water was used by the general population, inmates, and security personnel in Biratnagar.

To identify the causative agent of this epidemic, serum samples from 48 patients were obtained at Koshi Zonal Hospital, the largest government hospital in this zone. Hepatitis A virus RNA and IgM against hepatitis B virus core antigen was not detected in the 48 serum samples. Conversely, IgG, IgM, and IgA against HEV were detected in 47 (97.9%), 45 (94%), and 45 (94%) serum samples, respectively, and HEV RNA was detected in 42 (87.5%) of 48 serum samples, which indicated that the epidemic was caused by HEV.

A partial 412-nt sequence from open reading frame 2 corresponding to nt 5944–6355 of the HEV B1 genome (*4*) was obtained as reported (*5*). We obtained 40 HEV isolates from the 42 samples and sequenced partial 412-nt segments. All 40 HEV sequences from the epidemic in Biratnagar segregated into a cluster within genotype 1a (Figure). These sequences showed 99.8% nt identity with each other but only 90.8%–95.4% nt identity with other HEV isolates from Nepal and those from India, Bangladesh, Pakistan, and China.

**Figure:**
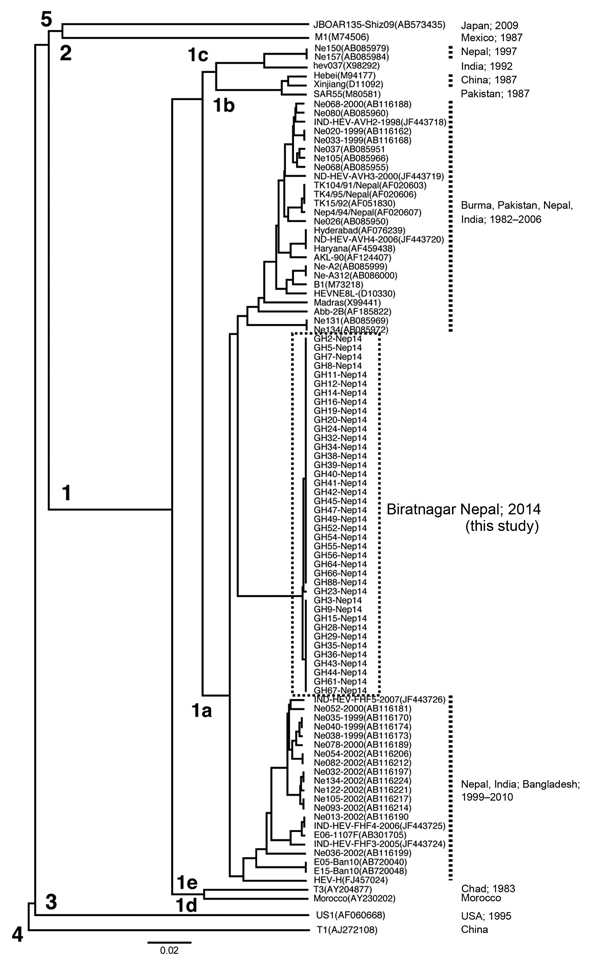
Phylogenetic analysis (UPGMA) of hepatitis E virus (HEV) sequences from the epidemic in Biratnagar, Nepal, 2014, on the basis of 412 nt within open reading frame 2 (DDBJ/EMBL/GenBank accession nos. AB98608–AB986107). All 40 HEV isolates from epidemic in Biratnagar segregated into a single, new cluster within genotype 1a. Values along branches indicate genotypes. Scale bar indicates nucleotide substitutions per site.

Compared with previous HEV epidemics in Nepal (*6*) and other parts of the Indian subcontinent, the local government of Biratnagar and central government of Nepal took steps to contain the reported epidemic. Activities of public and private sectors in Biratnagar ended the epidemic in ≈12 weeks, and no new cases of acute jaundice have been reported in Biratnagar.

Persons in Biratnagar were given information regarding epidemics and ways to contain them. They were instructed by electronic media to use boiled water for consumption. It became clear that additional information regarding about maintaining water and sewerage systems during road construction and repair should also be provided. Because 14 patients died of HEV infection during this epidemic, more preparedness for epidemics of waterborne diseases is required to minimize unnecessary illnesses and deaths.
